# 

**Published:** 1879-10-20

**Authors:** 


### TABLE OF CONTENTS.

ORIGINAL AND SELECTED ARTICLES.

Sexual Hypochondriasis, by F. J. Bumstead, M.D., 362. Wound to the Brain, by Thos. O. Summers, M.D., of Nashville, Tennessee, 366. Diseases of the Skin, by L. Duncan Bulkley, A.M., M.D., New York, 368. Electro-Therapeutics, by William F. Hutchinson, A.M., M.D., Providence, R. I., 373. Gonorrhœa, by Dr. H M. Lawson, of Georgia, 374. Preventing Mammary Abscess, by Francis J. Shepherd, M.D., M.R., C.S., Eng., 375. Dressing Head Wounds, by A. G. Smyth, M.D., of Miss., 376.

ABSTRACTS AND GLEANINGS.

Galvanic Current in Exophthalmic Goitre, 377. Appearance of the Tongue in Diseases, 378. Memoranda for the Disinfection of Yellow Fever, 380. Martin's Bandage in Chronic Ulcers, 381. Habitual Constipation—Its Cure, 382. Jaborandi in Cholera; Iodoform, 383. The Aspirator in Hernia; When to Induce Premature Labor in Puerperal Eclampsia, 384. Scilline, etc., the Active Principles of Squill, 385. External Application of the Bromide of Potassium; Esmarch's Tourniquet in the Treatment of Neuralgia, 386. Cholagogues; Hypodermic Injections of Chloral in Convulsions, 387. Incontinence of Urine in Children; Interstitial Pneumonia; Subcutaneous Injection of Ergot in Neuralgia, 388. Mammary Inflammation Treated by Ice; Poisoning by Carbolic Acid; The Choice of Purgatives, 389. Dentitition as a Cause of Diarrhœa; Dr. Robert's list of pathological States; A Urine Thermometer, 390.

SCIENTIFIC ITEMS.

The Cause of Thunder; Solar Systems Other Than Our Own, 391. Making the Deaf Hear; The Electric Light; A Milk Test, 392.

PRACTICAL NOTES AND FORMULÆ.

Cholera Infantum; Calomel, 393. How to Prevent Mammary Abscess; Dropsical Affections; Rheumatic Iritis; Painful Diarrhœa, etc.; Salicylic Acid in Ear Diseases, 394. Feted Discharges from Tooth Abscess Opening into the Antrum; Rapid Cure of Coryza; Gonorrhœa; Laxative Bread, 395. Pertussis; Burns; Nursing Sore Mouth; Salicylic Acid as an Anti-Scorbutic; Cholera Morbus; Hemorrhage from the Nose. 396.

EDITORIAL AND MISCELLANEOUS.

Editorial Notices; The Southern Medical College, 397. Disreputable; American Public Health Association; College Museum and Library; Book Notices, 398. Pamphlets Received, 399. Obituary; Receipted; Special Notices, 400.

